# Prognosis of resected non-small cell lung cancer with pleural plaques on intrathoracic findings

**DOI:** 10.1186/s12885-022-09600-6

**Published:** 2022-04-28

**Authors:** Atsushi Kagimoto, Takeshi Mimura, Atsushi Kamigaichi, Yoshinori Yamashita

**Affiliations:** Department of General Thoracic Surgery, National Hospital Organization, Kure Medical Center and Chugoku Cancer Center, 3-1 Aoyama-cho, Kure, Hiroshima, 737-0023 Japan

**Keywords:** Pleural plaques, Non-small cell lung cancer, Asbestos, Lung cancer

## Abstract

**Background:**

The prognosis of patients with lung cancer who demonstrate pleural plaques intraoperatively, which may be associated with exposure to asbestos, is unclear. Here, we compared the clinicopathological characteristics and prognosis of these patients to those of patients without pleural plaques.

**Methods:**

We included patients who underwent curative-intent resection for non-small cell lung cancer. We retrospectively investigated the relationship of intrathoracic findings of pleural plaques with clinicopathological features and prognosis.

**Results:**

Pleural plaques were found in 121/701 patients (17.3%) during surgery. The incidence of squamous cell carcinoma (*P* < 0.001) and the pathological stage (*P* = 0.021) were higher in patients with pleural plaques. Overall survival was significantly worse in patients with pleural plaques (5-year rate; 64.5% vs. 79.3%; *P* < 0.001), and the same finding was noted in clinical stage I patients (5-year rate; 64.8% vs. 83.4%; *P* < 0.001). In multivariable analysis, the presence of pleural plaques was a significant predictor of overall survival in patients with clinical stage I (hazard ratio, 1.643; *P* = 0.036). In the analysis among patients with emphysema more severe than Goddard score 5 points or interstitial pneumonia, overall survival was significantly worse in those with pleural plaques than in those without pleural plaques (5-year rate; 66.3% vs. 49.5%; *P* < 0.001).

**Conclusions:**

Patients with non-small cell lung cancer who underwent resection and demonstrated pleural plaques intraoperatively had a significantly worse prognosis. It is important to recognize the presence of pleural plaques intraoperatively, and our findings will be useful in determining the treatment and follow-up strategy for such patients with lung cancer and pleural plaques on intrathoracic examination.

**Supplementary Information:**

The online version contains supplementary material available at 10.1186/s12885-022-09600-6.

## Background

Pleural plaques are one of the most common manifestations associated with asbestos exposure [1]. The prevalence of bilateral pleural plaques ranged from 1.2% (latency < 16 years) to 32.2% (latency ≥ 40 years) in a large study of construction, shipyard, and asbestos industry workers; the mean exposure and time since first exposure to asbestos were determinants of the prevalence of pleural plaques [2]. Pleural plaques are irregularly distributed on the parietal and diaphragmatic pleura and may occasionally be identified on the visceral pleura. Pleural plaques also tend to follow the rib lines, often extending across one or more intercostal spaces [3]. Although pleural plaques are sometimes noted on preoperative chest computed tomography (CT) in patients with lung cancer who had been exposed to asbestos, general thoracic surgeons often encounter pleural plaques incidentally during surgery in patients who do not demonstrate pleural plaques on preoperative CT.

Asbestos can easily be released into the atmosphere and inhaled, leading to various diseases such as asbestosis [4] and mesothelioma [5]. Exposure to asbestos is also an acknowledged risk factor for primary lung cancer [6]. The global consumption of asbestos has been declining [7], and in Japan, the use of asbestos was banned in 2004. However, there is a notable latency period between exposure to asbestos and the occurrence of lung cancer, and the longest reported period was 47 years [8]. Therefore, asbestos-related lung cancer remains an important concern. Although there have been several etiological studies on asbestos-related lung cancer [8–10], there have been no reports on the prognosis of patients with resected lung cancer who have been exposed to asbestos.

Regarding the pathogenesis of pleural plaques as noted by Hillerdal [11], inhaled short asbestos fibers spread towards the visceral pleura of the lung, and some may penetrate the pleural space. There, these fibers follow the normal lymph flow from the pleural space, which is exclusively through the parietal pleura. Finally, they pass through the parietal pleura and cause a low-grade stimulation of the submesothelial fibroblasts, resulting in visible pleural plaques after a few decades. Therefore, pleural plaques are a reliable marker of past exposure to asbestos.

Kure in Hiroshima prefecture, Japan, where our facility is located, flourished as a naval port [12], and currently has a thriving shipbuilding industry. Therefore, we often encounter patients who have been exposed to asbestos as well as those with pleural plaques during surgery for lung cancer. In this study, we investigated the clinicopathological characteristics and prognosis of patients with non-small-cell lung cancer (NSCLC) who had intrathoracic findings of pleural plaques, and we compared these patients to those without pleural plaques.

## Methods

### Patients

**Fig. 1:**
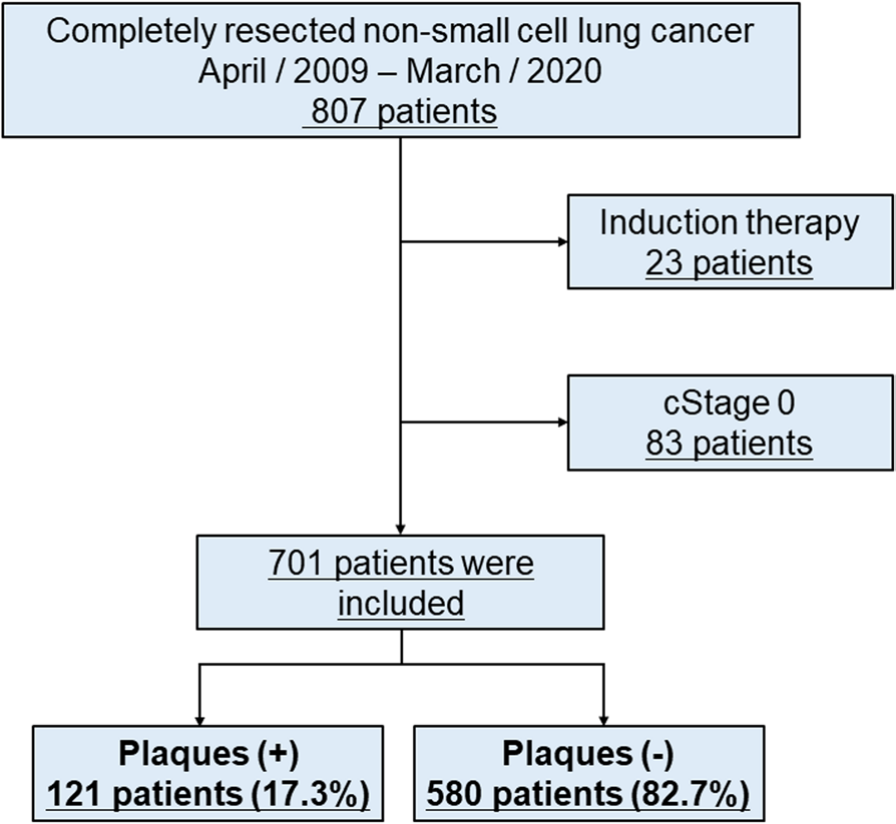
The flow chart of included patients. Totally, 701 patients were included in this study. Patients who underwent induction therapy, who could not achieve complete resection, and patients with clinical stage 0 were excluded from this study

This retrospective study was approved by the Institutional Review Board of our institute (The institutional review boards at Kure Medical Center and Chugoku Cancer Center, registration number: No. 2020–88). Because this is a retrospective study, it was not practical to obtain informed consent from all patients; therefore, patient consent was obtained using informed consent documents with an opt-out process. The study was carried out in accordance with institutional guidelines which were established based on the Declaration of Helsinki. The present study included patients who underwent curative-intent resection for NSCLC between April 2009 and March 2020 at the National Hospital Organization, Kure Medical Center, and Chugoku Cancer Center. We excluded patients who underwent induction therapy and for whom complete resection was not achieved. We also excluded patients with clinical stage 0 NSCLC because follow-up time from detection to resection varied among patients with pure ground-glass nodule, and their prognosis is known to be extremely good [13]. Figure 1 shows the patient selection scheme.

### Preoperative examinations

Chest CT, whole-body positron emission tomography with [^18^F]-fluoro-2-deoxy-D-glucose (FDG-PET/CT), brain magnetic resonance imaging (MRI), and pulmonary function tests were performed preoperatively to determine the clinical stage and treatment strategies. Clinical stage was determined based on the 8th edition of the TNM classification of malignant tumors [14]. Clinical N stage was radiologically diagnosed (enlargement > 1 cm on preoperative CT or significant accumulation of FDG on PET/CT). When clinical stage was difficult to diagnose radiologically, mediastinal diagnosis (lymph node biopsy by mediastinal scope or endobronchial ultrasound-guided trans-bronchial needle aspiration) was considered to help determine surgical indications and procedures. Interstitial pneumonia (IP) was diagnosed based on the radiological findings according to the guidelines of the American Thoracic Society, European Respiratory Society, Japanese Respiratory Society, and Latin American Thoracic Association [15]. The radiological emphysematous changes were diagnosed and evaluated using the Goddard score (GS) [16]. To evaluate GSs, the entire lung field was divided into three areas: the upper (level of the aortic arch), middle (level of the carina), and lower (level of the upper end of the diaphragm), with a total of six areas including both sides. Thereafter, the severity of emphysema was evaluated in each area. Areas without emphysematous changes were scored as 0 points. Emphysematous changes extending to < 25% of the lung field were scored as 1 point, ≥ 25%—< 50% were scored as 2 points, ≥ 50%—< 75% were scored as 3 points, and ≥ 75% were scored as 4 points. The GS values in all six areas were evaluated and summed; the maximum possible score was 24 points.

### Surgical procedure

At our institution, lobectomy is considered initially, but sublobar resection is performed for patients with tumors < 2 cm, those with tumors with low metabolic activity, and those deemed intolerant to lobectomy. The decision regarding the selection of sublobar procedure (i.e., segmentectomy or wedge resection) is usually based on a combination of patient performance status, tumor characteristics, and surgeon preference. Wedge resection is preferred for small, pleural-based tumors, particularly for patients with poor performance status and comorbidities, whereas segmentectomy is usually required for large tumors that settle within a resectable anatomical segment. Generally, we performed surgery under complete video-assisted thoracoscopic surgery.

### Determining the presence of pleural plaques

**Fig. 2:**
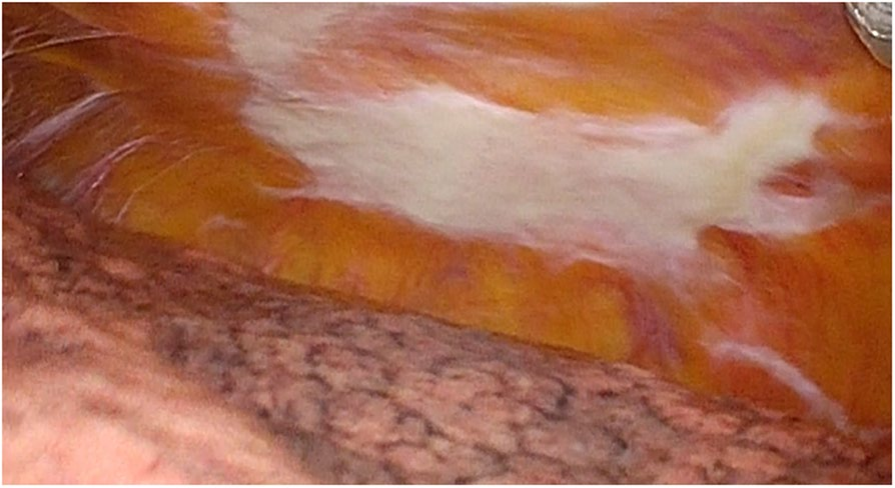
Representative image of pleural plaques. The presence of pleural plaque was judged by intraoperative examination of the thoracic cavity. We considered pleural plaques that extended beyond multiple intercostal spaces to be positive findings

The presence of pleural plaques was determined based on intraoperative findings in the thoracic cavity. We considered pleural plaques that extended beyond intercostal spaces to be positive findings. A similar degree of pleural plaques on the diaphragm was also considered positive. The presence of the plaques was confirmed by all the surgical staff and was prospectively recorded in our database. Furthermore, all surgical videos were stored. A representative image of a pleural plaque is shown in Fig. 2.

### Pathological examinations

Pathological staging of lung cancer was determined based on the 8^th^ edition of the TNM classification of malignant tumors [14]. The histological diagnosis was determined based on the World Health Organization classification [17]. All patients were evaluated for lymphatic invasion, vascular invasion, and pleural invasion. The diagnosis of lymphatic invasion was based on pathological examination using immunostaining for D2-40 to clarify the location of the lymphatic duct. The presence of pleural and vascular invasion was evaluated using elastic van Gieson staining to determine the degree of tumor invasion into the elastic layer of the vessels and visceral pleura.

### Follow-up evaluation

Postoperative follow-up procedures, including physical examination and chest CT scans, were performed every 6 months for 5 to 10 years after surgical resection. In cases where recurrence was suspected based on CT findings or symptoms, FDG-PET/CT and brain MRI were performed. Adjuvant therapy using a platinum agent was considered when pathological lymph node metastasis was present. Adjuvant therapy using an oral anticancer agent was considered when the pathological tumor size was > 20 mm. However, the decision to perform postoperative adjuvant therapy or not was ultimately made by the attending surgeon and the patient’s preferences.

### Statistical analyses

The results are presented as the median and interquartile range (IQR) for continuous variables, and as number (n) and percentage for categorical variables. Recurrence-free survival (RFS) was defined as the time interval from the date of surgery until the date of recurrence, death, or the last follow-up visit. Overall survival (OS) was defined as the time interval from the date of surgery until the date of death due to any cause or until the last follow-up visit. Survival data were calculated using the Kaplan–Meier method and compared using the log-rank test. Categorical variables were compared using the chi-square test. Continuous variables were analyzed using an unpaired t-test. We calculated the incidence of death from respiratory disease, which was defined as death due to bacterial pneumonia, fungal pneumonia, interstitial pneumonia, emphysema, and respiratory diseases other than lung cancer. Respiratory diseases other than lung cancer-specific survival (RSS) and lung cancer-specific survival (CSS) were also calculated using the Kaplan–Meier method and compared using the log-rank test. Univariable and multivariable analyses using Cox proportional hazards regression analysis for OS were also performed for patients with clinical stage I. In the multivariable analysis, the patients’ background characteristics such as age (≥ 70 years or < 70 years), sex, radiological IP findings, radiological emphysematous change with GS ≥ 5 points, solid tumor size (> 30 mm / ≤ 30 mm), and presence of pleural plaques were included as variables.

JMP® version 14 (SAS Institute, Cary, NC, USA) was used for all statistical analyses. *P* < 0.05 was considered statistically significant.

## Results

**Fig. 3:**
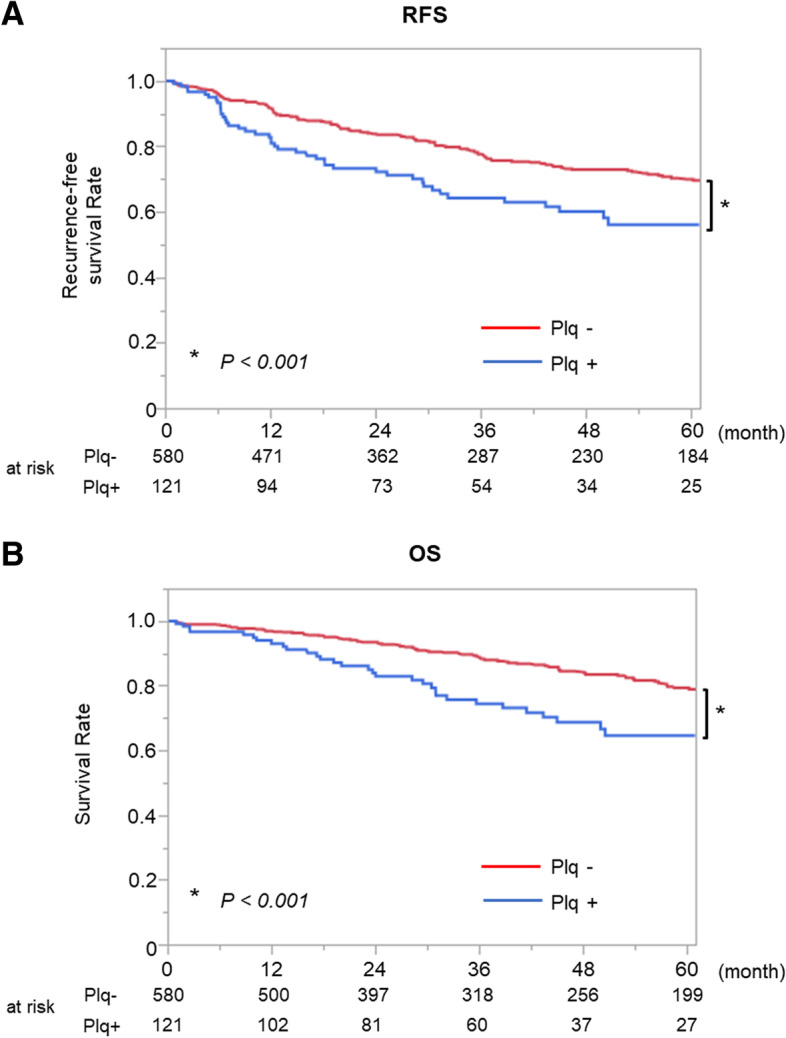
Prognosis of all included patients. **A** Recurrence free survival (RFS) was significantly worse in patients with pleural plaques (5-year RFS rate: 56.1%; 95% confidence interval [CI], 45.3%–66.3%) than in those without pleural plaques (70.0%; 95% CI, 65.2%–74.3%; *P* < 0.001). **B** Overall survival (OS) was significantly worse in patients with pleural plaques (5-year OS rate: 64.5%; 95% CI, 53.2%–74.4%) than in those without pleural plaques (79.3%; 95% CI, 74.7%–83.2%; *P* < 0.001)

We included 701 patients in this study, and the median follow-up period was 43 (IQR, 18–73) months. Of the 701 patients, 123 (17.3%) were noted to have pleural plaques during the procedure. The patients’ characteristics according to the presence of pleural plaques are shown in Table 1. Older age (*P* < 0.001), male predominance (*P* < 0.001), history of smoking (*P* < 0.001), large whole tumor size (*P* = 0.024), and large solid component size (*P* = 0.013) were characteristics of patients with pleural plaques. However, there were no significant differences in clinical stage (*P* = 0.561) or surgical procedure (*P* = 0.676) between these patients and those without pleural plaques. The maximum standardized uptake value (SUVmax) in FDG-PET/CT (*P* < 0.001), the incidence of squamous cell carcinoma (*P* < 0.001), and the pathological stage (*P* = 0.021) were higher in patients with pleural plaques than in those without pleural plaques. The incidences of radiological emphysematous changes with GS ≥ 5 points (*P* < 0.001) and radiological IP findings (*P* = 0.004) in preoperative CT were also higher in patients with pleural plaques. RFS was significantly worse in patients with pleural plaques (5-year RFS rate, 56.1%; 95% confidence interval [CI], 45.3–66.3%) than in those without pleural plaques (70.0%; 95% CI, 65.2–74.3%; *P* < 0.001) (Fig. 3A). Also, there were no patients who developed malignant pleural mesothelioma after surgery for lung cancer in our cohort. OS was also significantly worse in patients with pleural plaques (5-year OS rate: 64.5%; 95% CI, 53.2–74.4%) than in those without pleural plaques (79.3%; 95% CI, 74.7–83.2%; *P* < 0.001) (Fig. 3B). Although there were no significant differences in the incidences of recurrence (*P* = 0.192) or death from lung cancer (*P* = 0.115), the incidence of death from respiratory diseases other than lung cancer was higher in patients with pleural plaques (*P* = 0.001). The details of death from respiratory diseases are shown in Supplemental Table 1. The details of death from causes other than respiratory disease and lung cancer are also shown in Supplemental Table 2. RSS (*P* < 0.001; Supplemental Figure 1A) and CSS (*P* = 0.026; Supplemental Figure 1B) were also shorter in patients with pleural plaques.

**Table 1:** Characteristics of all included patients

Variables	Plaques ( +) *n* = 121 (17.3%)	Plaques (-) *n* = 580 (82.7%)	*P* value
Age (median, IQR)	74 (70–78)	72 (65–77)	< 0.001
Sex, Male	112 (92.5%)	313 (54.0%)	< 0.001
Smoking history	111 (91.7%)	342 (59.0%)	< 0.001
Preoperative CT findings			
Radiological emphysematous changes (GS ≥ 5 points)	54 (44.6%)	138 (23.8%)	< 0.001
Radiological IP findings	39 (32.2%)	115 (19.8%)	0.004
Whole tumor size (mm) (median, IQR)	21 (15–31)	20 (14–28)	0.024
Solid tumor size (mm) (median, IQR)	19 (12–28)	15 (10–25)	0.013
SUVmax	5.4 (2.4–13.0)	2.6 (1.3–6.3)	< 0.001
Pulmonary function			
%VC (%) (median, IQR)	95.9 (86.4–109.7)	102.5 (89.7–113.8)	0.010
FEV1/FVC (%) (median, IQR)	75.8 (67.2–85.3)	77.8 (71.6–85.6)	0.007
Clinical stage			0.561
IA1	24 (19.8%)	153 (26.3%)	
IA2	41 (33.9%)	188 (32.4%)	
IA3	22 (18.2%)	92 (15.8%)	
IB	15 (12.4%)	64 (11.0%)	
IIA	3 (2.5%)	10 (1.7%)	
IIB	11 (9.1%)	41 (7.1%)	
IIIA	5 (4.1%)	25 (4.3%)	
IIIB	0 (0%)	7 (1.2%)	
Surgical procedure			0.676
Wedge resection	31 (25.6%)	180 (31.0%)	
Segmentectomy	17 (14.1%)	78 (13.4%)	
Lobectomy	71 (58.7%)	315 (54.3%)	
Pneumonectomy	2 (1.7%)	7 (1.2%)	
Invasive characteristics			
LY	14 (11.6%)	57 (9.8%)	0.569
V	13 (10.7%)	54 (9.3%)	0.631
PL			0.249
PL1	13 (10.7%)	49 (8.5%)	
PL2	3 (2.5%)	14 (2.4%)	
PL3	7 (5.8%)	14 (2.4%)	
Histology			< 0.001
Adenocarcinoma	66 (54.5%)	460 (79.3%)	
Squamous cell carcinoma	47 (38.8%)	91 (15.7%)	
Adenosquamous carcinoma	2 (1.7%)	14 (2.4%)	
Sarcomatoid carcinoma	1 (0.8%)	6 (1.0%)	
LCNEC	4 (3.3%)	7 (1.2%)	
Lymphoepithelioma-like carcinoma	1 (0.8%)	1 (0.2%)	
Mucoepidermoid carcinoma	0 (0%)	1 (0.2%)	
Pathological stage			0.021
0	5 (4.1%)	49 (8.4%)	
IA1	26 (21.5%)	171 (29.5%)	
IA2	24 (19.8%)	113 (19.5%)	
IA3	16 (13.2%)	74 (12.8%)	
IB	20 (16.5%)	76 (13.1%)	
IIA	5 (4.1%)	8 (1.4%)	
IIB	10 (8.3%)	50 (8.6%)	
IIIA	15 (12.4%)	32 (5.5%)	
IIIB	0 (0%)	7 (1.2%)	
Recurrence	28 (23.1%)	104 (17.9%)	0.192
Death from any cause	37 (30.6%)	91 (15.7%)	< 0.001
Death from lung cancer	16 (13.2%)	49 (8.5%)	0.115
Death from respiratory disease	12 (9.9%)	21 (3.6%)	0.007
Death from causes other than lung cancer and respiratory disease	9 (7.4%)	21 (3.6%)	0.079

*IQR* interquartile range, *CCI* Charlson comorbidity index, *CT* computed tomography, *IP* interstitial pneumonia, *GS* Goddard score, *SUV* maximum standardized uptake value, *VC* vital capacity, *FEV1.0* forced expiratory volume in one second, *LY* lymphatic invasion, *V* vascular invasion, *PL* pleural invasion, *LCNEC* large cell neuroendocrine carcinoma

**Fig. 4:**
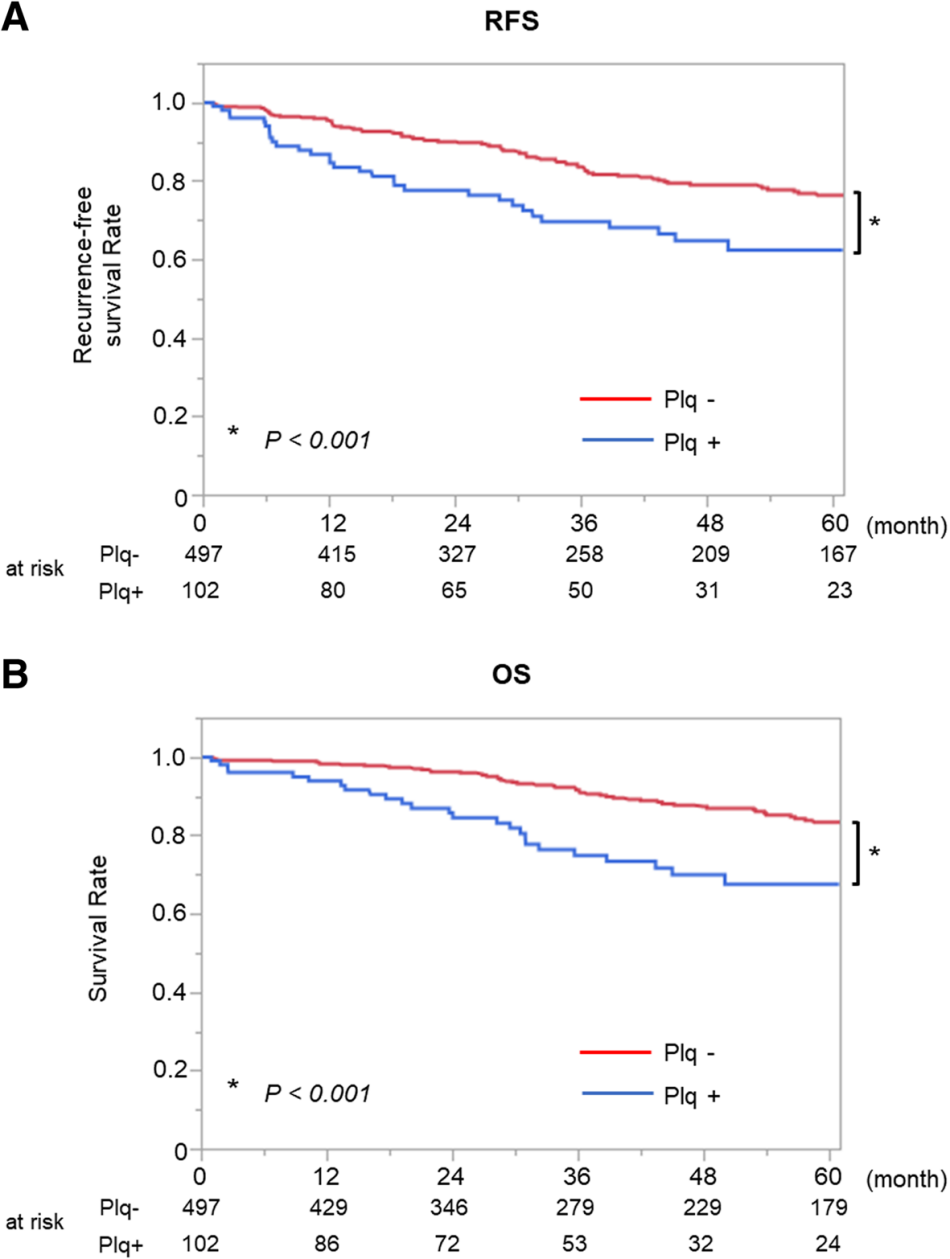
Prognosis of patients with clinical stage I. **A** RFS was significantly worse in patients with pleural plaques (5-year RFS rate: 62.5%; 95% CI, 50.9%–72.7%) than in those without pleural plaques (76.4%; 95% CI, 71.5%–80.7%; *P* < 0.001). **B** OS was also significantly worse in patients with pleural plaques (5-year OS rate: 64.8%; 95% CI, 52.2%–75.7%) than in those without pleural plaques (83.4%; 95% CI, 78.7%–87.2%; *P* < 0.001)

In the analysis limited to clinical stage I, which aimed to eliminate the effect of the cancer stage, the patient characteristics were similar to those of the entire study population (Supplemental Table 3). Among patients with clinical stage I, both RFS and OS were significantly worse in patients with pleural plaques (5-year RFS rate, 62.5% [95% CI, 50.9–72.7%]; 5-year OS rate, 64.8% [95% CI, 52.2–75.7%]) than in those without pleural plaques (5-year RFS rate, 76.4% [95% CI, 71.5–80.7%]; *P* < 0.001; 5-year OS rate, 83.4% [95% CI, 78.7–87.2%]; *P* < 0.001) (Fig. 4A and B). As with the entire study population, although there were no significant differences in the incidences of recurrence (*P* = 0.215) or death from lung cancer (*P* = 0.164), the incidence of death from respiratory diseases other than lung cancer was higher in patients with pleural plaques (*P* = 0.002). RSS (*P* < 0.001, Supplemental Figure 2A) and CSS (*P* = 0.044, Supplemental Figure 2B) were also shorter in patients with pleural plaques.

In the univariable analysis of patients with clinical stage I, the presence of pleural plaques was a significant predictor of OS (hazard ratio [HR], 2.984; 95% CI, 1.916–4.648; *P* < 0.001) and RFS (HR, 2.267; 95% CI, 1.545–3.326; *P* < 0.001). In the multivariable analysis, the presence of pleural plaques was also a significant predictor of OS (HR, 1.643; 95% CI, 1.033–2.615; *P* = 0.036) (Table 2).

**Table 2:** Univariable and multivariable analysis for OS in patients with clinical stage I

	Univariable analysis		Multivariable analysis	
Variables	HR (95% CI)	*P* value	HR (95% CI)	*P*-value
Age (≥ 70 / < 70)	2.536 (1.553–4.141)	< 0.001	1.931 (1.172–3.179)	0.010
Sex (male / female)	4.706 (2.661–8.325)	< 0.001	2.237 (1.186–4.220)	0.013
Radiological emphysematous changes (GS ≥ 5 points)	4.564 (3.013–6.913)	< 0.001	1.823 (1.134–2.932)	0.013
Radiological IP findings	5.360 (3.514–8.178)	< 0.001	3.127 (1.964–4.976)	< 0.001
Solid tumor size (> 30 mm / ≤ 30 mm)	2.644 (1.492–4.686)	0.001	2.422 (1.311–4.471)	0.005
Procedure (sublobar resection / lobectomy)	0.509 (0.332–0.780)	0.002	0.519 (0.330–0.817)	0.005
Presence of pleural plaques	2.984 (1.916–4.648)	< 0.001	1.643 (1.033–2.615)	0.036

*HR* hazard ratio, *CI* confidence interval, *GS* Goddard score, *IP* interstitial pneumonia

**Fig. 5:**
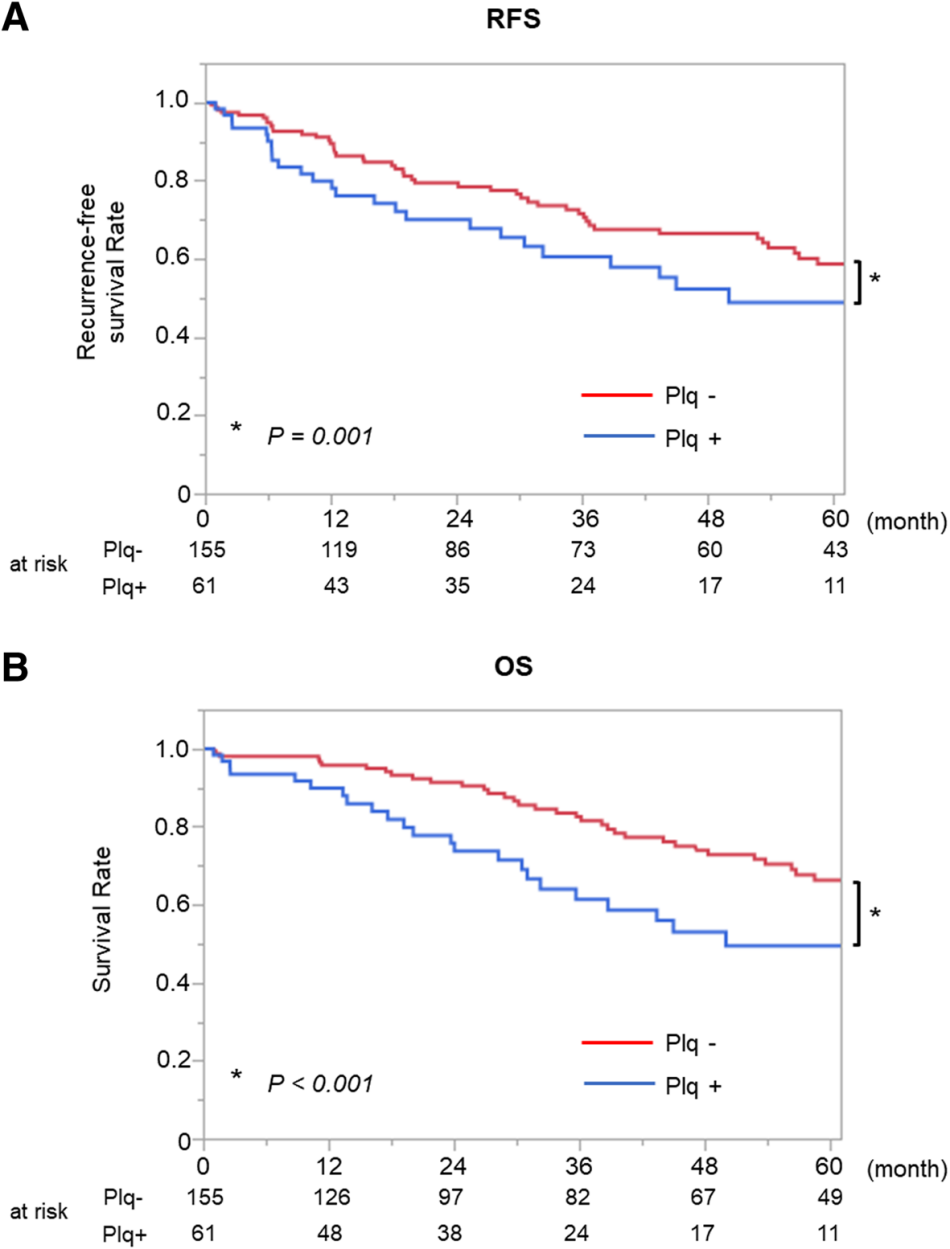
Prognosis of clinical stage I patients with emphysema with Goddard score ≥ 5 or interstitial pneumonia. **A** RFS of patients with pleural plaques (5-year RFS rate: 48.9%; 95% CI, 34.3%–63.7%) was worse than those of patients without pleural plaques (58.7%; 95% CI, 48.8%–68.0%; *P* = 0.001). **B** OS of patients with pleural plaques (5-year OS rate: 49.5%; 95% CI, 34.5%–64.6%) was worse than those of patients without pleural plaques (66.3%; 95% CI, 56.2%–75.1%; *P* < 0.001)

In the analysis of clinical stage I patients with radiological emphysematous changes with GS ≥ 5 points or IP (characteristics are shown in Supplemental Table 4), RFS (*P* = 0.001; Fig. 5A) and OS (*P* < 0.001; Fig. 5B) were shorter in patients with pleural plaques, even though there was no significant difference in either clinical or pathological stages. Although RSS was significantly worse in patients with pleural plaques (5-year RSS rate, 82.0%; 95% CI, 64.7–91.9%) than in those without pleural plaques (5-year RSS rate, 90.1%; 95% CI, 81.5–95.0%; *P* < 0.001; Supplemental Figure 3A), there was no significant difference in CSS between patients with pleural plaques (5-year CSS rate, 75.0%; 95% CI, 59.0–86.3%) and without pleural plaques (5-year CSS rate, 80.2%; 95% CI, 70.7–87.2%; *P* = 0.287; Supplemental Figure 3B).

## Discussion

In our study, the prognosis of patients with NSCLC who underwent resection and demonstrated pleural plaques intraoperatively was worse than that of patients without pleural plaques. This finding was also noted in the analysis limited to patients with clinical stage I NSCLC. In the overall population, lung cancer in patients with pleural plaques had more invasive features than that in patients without pleural plaques; the pathological stage was more advanced and squamous cell carcinomas were more common. Moreover, there were more deaths due to respiratory diseases among patients with pleural plaques. These facts might have led to a worse prognosis in patients with NSCLC patients who had pleural plaques. To our knowledge, the present study is the first to investigate the prognosis of patients with NSCLC who underwent resection and had pleural plaques intraoperatively.

In the present study, we focused on the presence or absence of intrathoracic findings of pleural plaques, rather than the often-reported pleural plaques on CT scans [1, 2, 18]. In a previous study by Pairon et al. [18], 1,118 (20.7%) of 5,402 retired male workers with a history of occupational exposure to asbestos presented with parietal pleural plaques with or without diaphragmatic pleural plaques on chest CT. The authors reported an adjusted HR for lung cancer of 2.41 (95% CI, 1.21–4.5) with the presence of pleural plaques. Pleural plaques are often noted on a preoperative chest CT scan in patients with lung cancer who had been exposed to asbestos, but general thoracic surgeons occasionally encounter the pleural plaques incidentally during surgery in those who do not demonstrate pleural plaques on a preoperative CT scan. We particularly conducted this study because we frequently encounter pleural plaques during thoracic surgery, as our facility is located in a city with a thriving shipbuilding industry.

Pleural plaques are irregularly distributed on the parietal and diaphragmatic pleura and may occasionally be identified on the visceral pleura. Pleural plaques also tend to follow rib lines, often extending across one or more intercostal spaces [3]. We considered pleural plaques that extended beyond multiple intercostal spaces to be positive findings, and pleural plaques were found in 17.3% of our study population. The presence or absence of asbestos exposure in each patient was not known in this study; however, pleural plaques were more frequently encountered intraoperatively in our hospital, which is located in an industrial area.

Asbestos exposure is an acknowledged risk factor for primary lung cancer [6]. Although there are several etiological studies about asbestos-related lung cancer [9, 10], there have been few reports on the prognosis of patients with lung cancer exposed to asbestos. In a previous study by Kishimoto et al., survival rates were similar between patients with asbestos-related lung cancer and those with non-asbestos-related lung cancer [8]. However, the study included patients with various stages of lung cancer, and therapeutic procedures varied considerably, including surgery, chemotherapy, and chemoradiotherapy. Alternatively, we examined a cohort in which all patients underwent complete resection for NSCLC. In the present study, patients with NSCLC who underwent resection and were identified with pleural plaques, which strongly suggests exposure to asbestos, had a worse prognosis after resection than those without pleural plaques. Although there were no significant differences in clinical stage, patients with pleural plaques demonstrated a higher SUVmax in FDG-PET/CT scans, which is known as a predictor of tumor invasiveness [19], higher pathological stage, and greater incidence of more invasive subtypes such as squamous cell carcinoma. Similar results were obtained in the analysis of patients with clinical stage I. Exposure to asbestos could contribute to the development of more invasive lung cancer. Oxidative DNA damage induced by asbestos is a reported cause of asbestos carcinogenesis [20]. Another study demonstrated that the interaction of lung fibroblasts with asbestos may support the growth and metastasis of lung cancer cells [21]. These factors may affect the invasiveness of NSCLC in patients with pleural plaques.

In our study, the incidence of death from respiratory diseases other than lung cancer was higher in patients with pleural plaques. Asbestos induces several respiratory diseases, including asbestosis and pleuritis [22]. Moreover, the presence of pleural plaques is associated with a small, but significant mean difference in forced vital capacity and forced expiratory volume in one second compared to asbestos-exposed individuals without plaques or other abnormalities [23]. These may contribute to the vulnerability of patients with pleural plaques to lung disease. In the multivariable analysis, the presence of pleural plaques was a significant predictor of worse OS. Among patients with emphysematous changes with GS ≥ 5 points [16] or IP [24], which are known as worse prognostic factors of NSCLC, OS was significantly shorter in patients with pleural plaques. These results indicate that the presence of pleural plaques independently affects the prognosis of patients with NSCLC.

There are several limitations to this study. First, the inherent limitations of a single-institution, retrospective study are recognized. Secondly, there was no information on individual exposure to asbestos, and thus, an analysis based on the history of exposure to asbestos could not be performed. Information on the presence of asbestos bodies in the lungs, fibrosis, and other pathological findings associated with asbestos would have been ideal. Third, there was also no information about the preoperative CT findings of pleural plaques. We investigated the outcomes in patients with pleural plaques at the time of lung cancer surgery in the present study, and we would like to examine the discrepancy between CT findings and intraoperative findings in a future study. Fourth, not only the incidence of respiratory disease, but also the incidence of death from causes other than respiratory disease and lung cancer was higher in patients with pleural plaques. The incidence of death from other cancers appeared to be higher in patients with pleural plaques, however, the reason for this is unknown. Finally, the presence of pleural plaques was confounded by other factors, such as sex and smoking. In addition, because oncological factors such as stage and histological type were among the outcomes, it was not possible to adjust for these factors with methods such as propensity score matching. However, the presence of pleural plaques on intrathoracic examination was a significant predictor of OS in the multivariable analysis containing other factors that could affect prognosis. This indicates that the presence of pleural plaque is independently significant from other factors.

Despite the above-mentioned limitations, our study seems important, as there have been no other studies on resected NSCLC with asbestos exposure or intrathoracic findings of pleural plaques. Most past studies [8–10] on asbestos exposure and lung cancer included patients who did not undergo surgery. Moreover, the prognoses of resected cases were completely unknown. The pathological diagnosis of our study was more accurate because we used surgical specimens, and we also confirmed the presence of pleural plaques intraoperatively. We believe that this study is worthwhile because patients with NSCLC and pleural plaques were observed to have a worse prognosis, and it is important to recognize the presence of pleural plaques intraoperatively. Specifically, the examination frequency could be increased or follow-up periods could be extended for patients with pleural plaques.

## Conclusion

Patients with NSCLC who underwent resection and were identified with pleural plaques intraoperatively had a significantly worse prognosis. Careful follow-up after resection is needed because these patients tend to have lung cancer with invasive characteristics and die from respiratory disease. It is important to recognize the presence of pleural plaques intraoperatively, and our findings will be useful in determining the treatment and follow-up strategy for patients with lung cancer who have pleural plaques or a history of exposure to asbestos.

## Supplementary Information

**Additional file 1: Figure S1.** Prognosis of all included patients.

**Additional file 2: Figure S2.** Prognosis of patients with clinical stage I cancer.

**Additional file 3: Figure S3.** Prognosis of clinical stage I patients with emphysematous change with Goddard score ≥5 points or interstitial pneumonia.

**Additional file 4: Table S1.** Detail of death from respiratory disease other than lung cancer.

**Additional file 5: Table S2.** Detail of death from other than respiratory disease and lung cancer.

**Additional file 6: Table S3.** Characteristics of patients with stage I.

**Additional file 7: Table S4.** Characteristics of clinical stage I patients with emphysema with Goddard score ≥5 points or interstitial pneumonia.

## Data Availability

All data generated or analyzed during this study are included in this published article.

## Abbreviations

CSS: Cancer specific survival
CT: Computed tomography
CI: Confidence interval
FDG: [18F]-fluoro-2-deoxy-D-glucose
GS: Goddard score
HR: Hazard ratio
IP: Interstitial pneumonia
IQR: Interquartile range
MRI: Magnetic resonance imaging
NSCLC: Non-small-cell lung cancer
OS: Overall survival
PET: Positron emission tomography
RFS: Recurrence-free survival
RSS: Respiratory disease other than lung cancer specific survival
SUV: Maximum standardized uptake value
