# Supplementary figures and images for: Prognosis of resected non-small cell lung cancer with pleural plaques on intrathoracic findings
Source: BMC Cancer. 2022 Apr 28;22:469. doi: 10.1186/s12885-022-09600-6 (PMC9052480; doi:10.1186/s12885-022-09600-6)

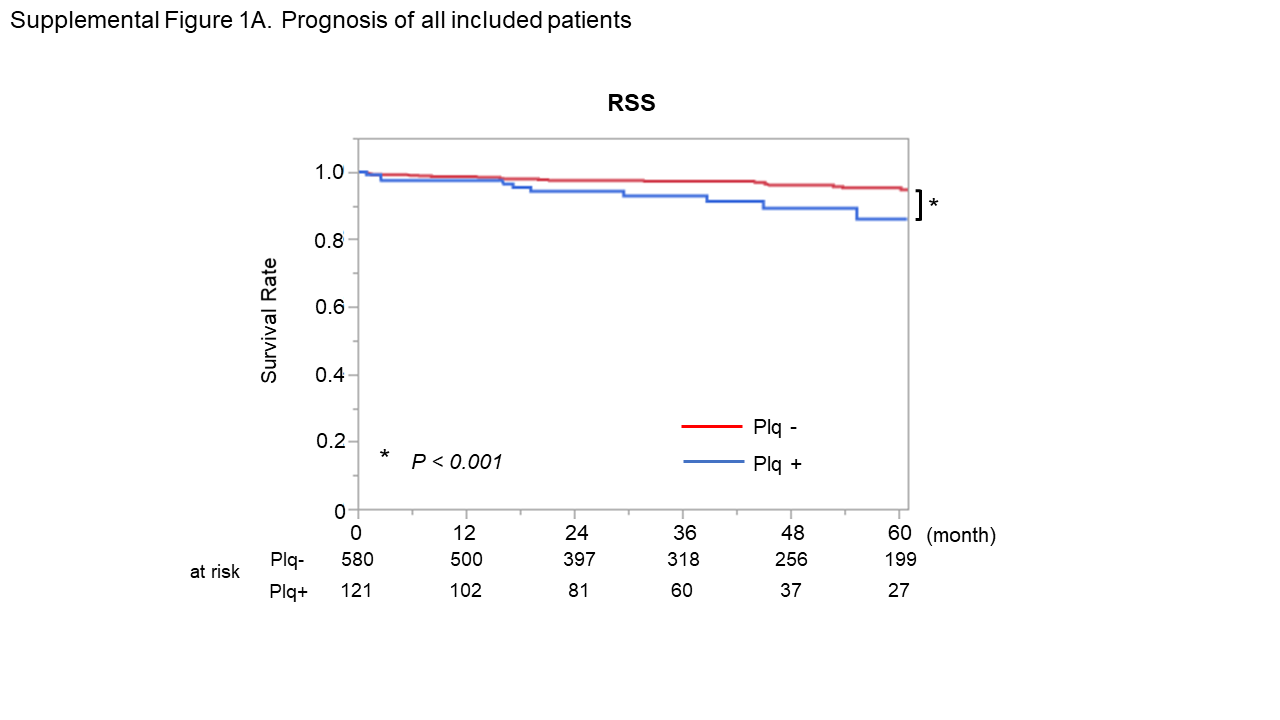

Supplement: Supplementary file 1 — Additional file 1: Figure S1. Prognosis of all included patients. [file 12885_2022_9600_MOESM1_ESM.zip › Supplemental Figure 1A.tif]

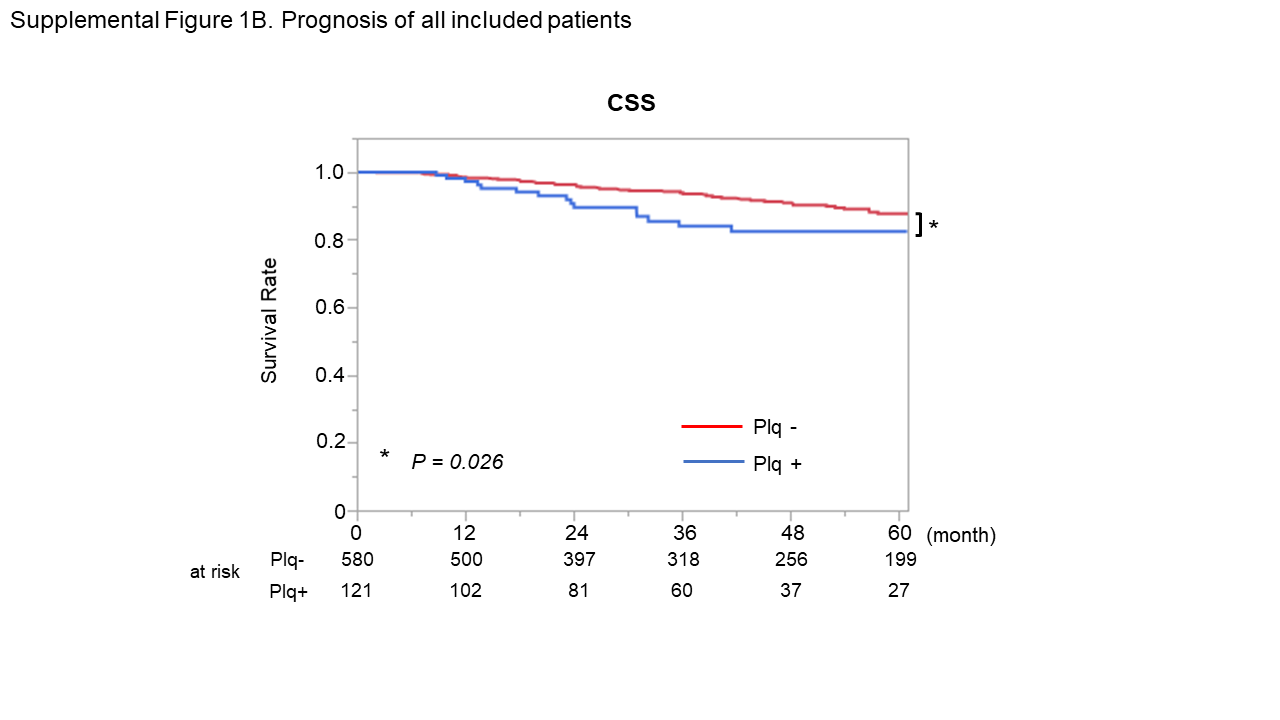

Supplement: Supplementary file 1 — Additional file 1: Figure S1. Prognosis of all included patients. [file 12885_2022_9600_MOESM1_ESM.zip › Supplemental Figure 1B.tif]

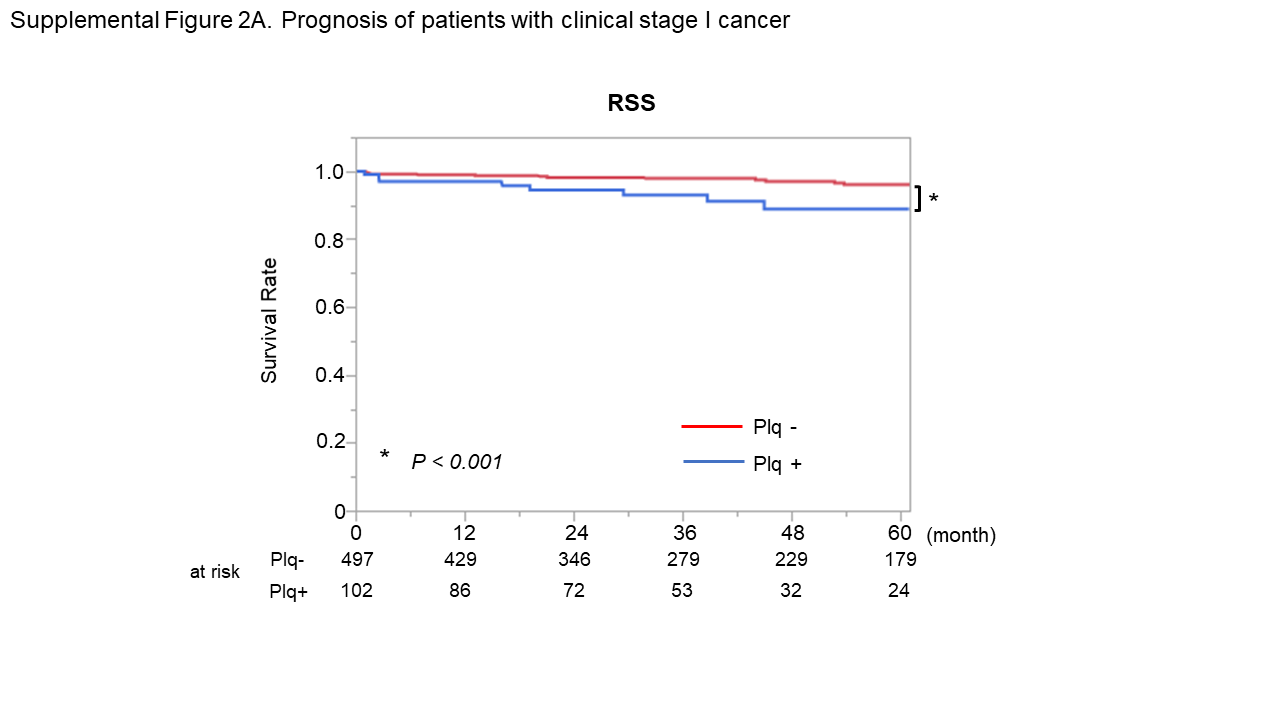

Supplement: Supplementary file 2 — Additional file 2: Figure S2. Prognosis of patients with clinical stage I cancer. [file 12885_2022_9600_MOESM2_ESM.zip › Supplemental Figure 2A.tif]

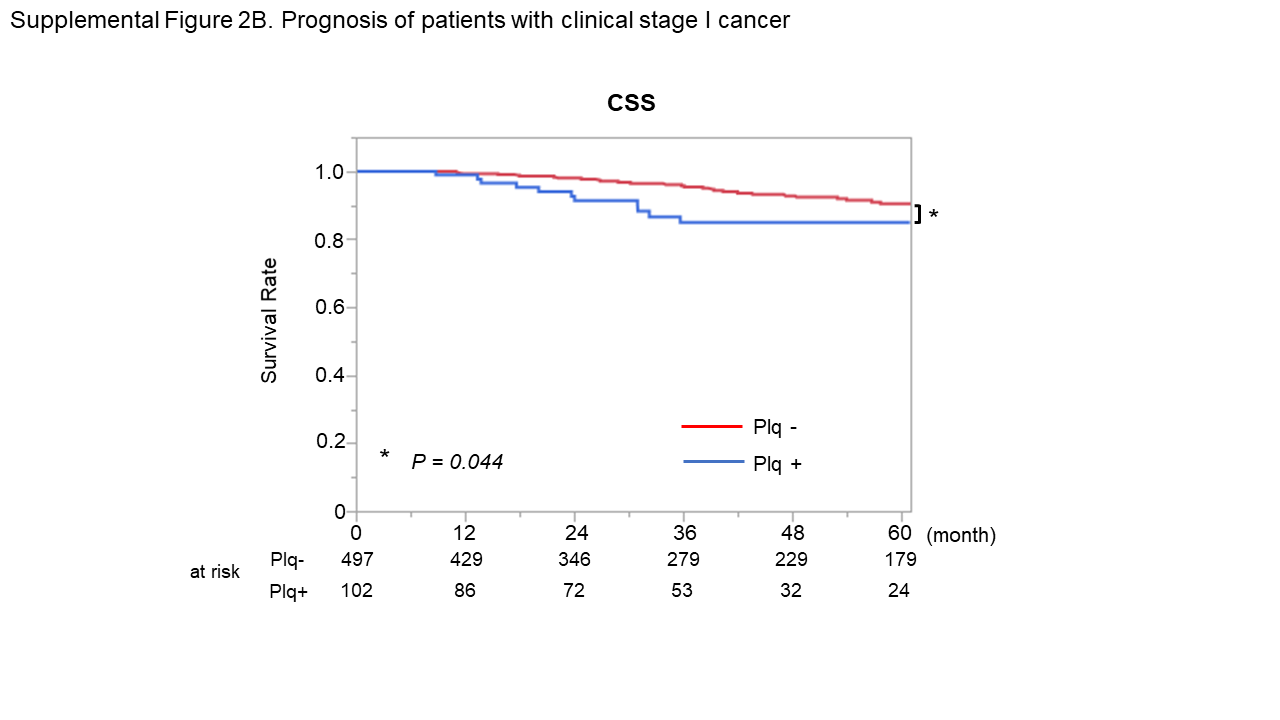

Supplement: Supplementary file 2 — Additional file 2: Figure S2. Prognosis of patients with clinical stage I cancer. [file 12885_2022_9600_MOESM2_ESM.zip › Supplemental Figure 2B.tif]

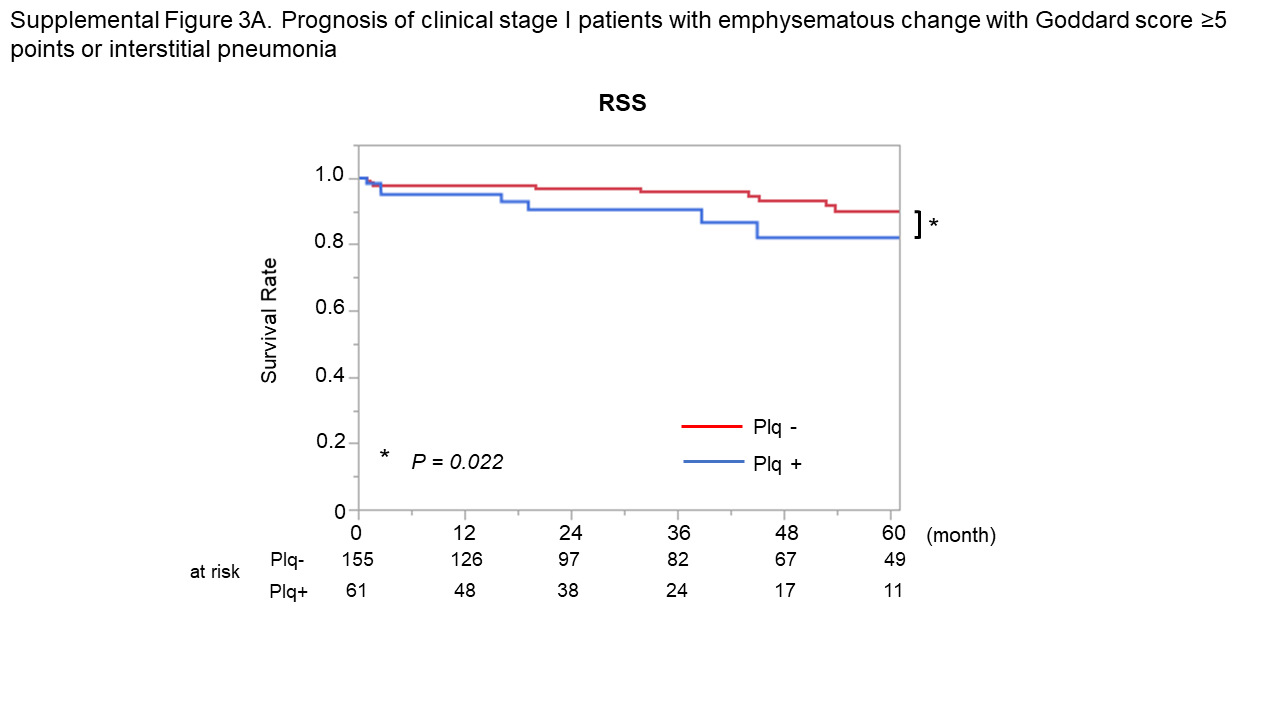

Supplement: Supplementary file 3 — Additional file 3: Figure S3. Prognosis of clinical stage I patients with emphysematous change with Goddard score ≥5 points or interstitial pneumonia. [file 12885_2022_9600_MOESM3_ESM.zip › Supplemental Figure 3A.tif]

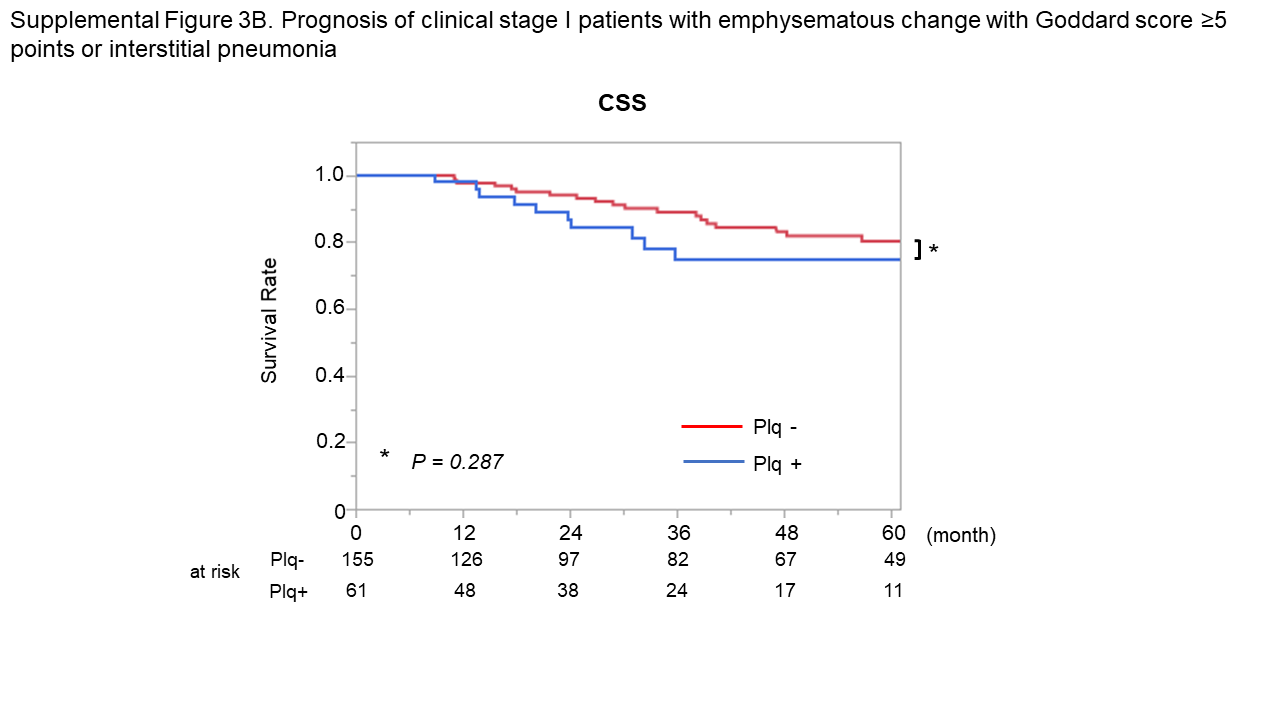

Supplement: Supplementary file 3 — Additional file 3: Figure S3. Prognosis of clinical stage I patients with emphysematous change with Goddard score ≥5 points or interstitial pneumonia. [file 12885_2022_9600_MOESM3_ESM.zip › Supplemental Figure 3B.tif]
