# Supplementary material for: Prognosis of resected non-small cell lung cancer with pleural plaques on intrathoracic findings
Source: BMC Cancer. 2022 Apr 28;22:469. doi: 10.1186/s12885-022-09600-6 (PMC9052480; doi:10.1186/s12885-022-09600-6)
Supplement: Supplementary file 4 — Additional file 4: Table S1. Detail of death from respiratory disease other than lung cancer. [file 12885_2022_9600_MOESM4_ESM.docx]

**Supplemental Table 1. Detail of death from respiratory disease other than lung cancer**

| Variables | Plaques (+)  n = 12 / 121 (9.9%) | Plaques (-)  n = 21 / 580 (3.6%) |
| --- | --- | --- |
| Bacterial pneumonia | 6 | 15 |
| Interstitial pneumonia | 3 | 5 |
| Fungal pneumonia | 3 | 0 |
| Empyema | 0 | 1 |
