# Supplementary material for: Prognosis of resected non-small cell lung cancer with pleural plaques on intrathoracic findings
Source: BMC Cancer. 2022 Apr 28;22:469. doi: 10.1186/s12885-022-09600-6 (PMC9052480; doi:10.1186/s12885-022-09600-6)
Supplement: Supplementary file 5 — Additional file 5: Table S2. Detail of death from other than respiratory disease and lung cancer. [file 12885_2022_9600_MOESM5_ESM.docx]

**Supplemental Table 2. Detail of death from other than respiratory disease and lung cancer**

| Variables | Plaques (+)  n = 9 / 121 (7.4%) | Plaques (-)  n = 21 / 580 (3.6%) |
| --- | --- | --- |
| Other cancer | 5 | 8 |
| Renal failure | 0 | 2 |
| Trauma | 1 | 1 |
| Pulmonary thromboembolism | 0 | 1 |
| Dilated cardiomyopathy | 0 | 1 |
| Aortic dissection | 0 | 1 |
| Heart failure | 0 | 1 |
| sigmoid volvulus | 0 | 1 |
| Sepsis | 0 | 1 |
| Subarachnoid Hemorrhage | 1 | 0 |
| Diverticulitis | 1 | 0 |
| Sudden death of unknown cause | 1 | 4 |
