# Supplementary material for: Prognosis of resected non-small cell lung cancer with pleural plaques on intrathoracic findings
Source: BMC Cancer. 2022 Apr 28;22:469. doi: 10.1186/s12885-022-09600-6 (PMC9052480; doi:10.1186/s12885-022-09600-6)
Supplement: Supplementary file 6 — Additional file 6: Table S3. Characteristics of patients with stage I. [file 12885_2022_9600_MOESM6_ESM.docx]

**Supplemental Table 3. Characteristics of patients with stage I**

| Variables | Plaques (+)  n = 102 (17.0%) | Plaques (-)  n = 497 (83.0%) | *P* value |
| --- | --- | --- | --- |
| Age (median, IQR) | 74 (70–80) | 72 (66–77) | < 0.001 |
| Sex, Male | 94 (92.1%) | 247 (49.7%) | < 0.001 |
| Smoking history | 92 (90.2%) | 274 (55.1%) | < 0.001 |
| Brinkman index (median, IQR) | 940 (590–1200) | 200 (0–900) | < 0.001 |
| Preoperative CT findings |  |  |  |
| Radiological emphysematous changes (GS ≥5 points) | 46 (45.1%) | 106 (21.3%) | < 0.001 |
| Radiological IP findings | 36 (35.3%) | 86 (17.3%) | < 0.001 |
| Whole tumor size (mm) (median, IQR) | 20 (14–25) | 19 (13–25) | 0.403 |
| Solid tumor size (mm) (median, IQR) | 16 (11–23) | 14 (8–22) | 0.131 |
| SUVmax | 4.5 (2.2–10.2) | 2.1 (1.2–4.7) | < 0.001 |
| Pulmonary function |  |  |  |
| %VC (%) (median, IQR) | 95.8 (86.0–110.5) | 103.4 (90.3–113.8) | 0.016 |
| FEV1/FVC (%) (median, IQR) | 76.6 (69.4–81.5) | 77.6 (71.8–82.0) | 0.054 |
| Clinical stage |  |  | 0.506 |
| IA1 | 24 (23.5%) | 153 (30.8%) |  |
| IA2 | 41 (40.1%) | 188 (37.8%) |  |
| IA3 | 22 (21.6%) | 92 (18.5%) |  |
| IB | 15 (14.7%) | 64 (12.9%) |  |
| Surgical procedure |  |  | 0.749 |
| Wedge resection | 31 (25.6%) | 246 (49.5%) |  |
| Segmentectomy | 17 (14.1%) | 76 (15.3%) |  |
| Lobectomy | 71 (58.7%) | 246 (49.5%) |  |
| Pneumonectomy | 2 (1.7%) | 1 (0.2%) |  |
| Invasive characteristics |  |  |  |
| LY | 12 (11.8%) | 37 (7.4%) | 0.166 |
| V | 8 (7.8%) | 32 (6.4%) | 0.612 |
| PL |  |  | 0.659 |
| PL1 | 10 (9.8%) | 36 (7.2%) |  |
| PL2 | 3 (2.9%) | 11 (2.2%) |  |
| PL3 | 2 (2.0%) | 5 (1.0%) |  |
| Histology |  |  | < 0.001 |
| Adenocarcinoma | 60 (58.8%) | 415 (83.5%) |  |
| Squamous cell carcinoma | 35 (34.3%) | 58 (11.7%) |  |
| Adenosquamous cell carcinoma | 2 (2.0%) | 13 (2.6%) |  |
| Sarcomatoid carcinoma | 0 (0%) | 4 (0.8%) |  |
| LCNEC | 4 (4.0%) | 5 (1.0%) |  |
| Lymphoepithelioma-like carcinoma | 1 (0.8%) | 1 (0.2%) |  |
| Mucoepidermoid carcinoma | 0 (0%) | 1 (0.2%) |  |
| Pathological stage |  |  | 0.018 |
| 0 | 5 (4.9%) | 49 (9.9%) |  |
| IA1 | 26 (25.5%) | 170 (34.2%) |  |
| IA2 | 22 (21.6%) | 111 (22.3%) |  |
| IA3 | 16 (15.7%) | 66 (13.3%) |  |
| IB | 19 (18.6%) | 67 (13.5%) |  |
| IIA | 4 (3.9%) | 2 (0.4%) |  |
| IIB | 6 (5.9%) | 26 (5.2%) |  |
| IIIA | 4 (3.9%) | 6 (1.2%) |  |
| Recurrence | 18 (17.7%) | 64 (12.9%) | 0.215 |
| Death from any cause | 30 (29.4%) | 60 (12.7%) | < 0.001 |
| Death from lung cancer | 11 (10.8%) | 33 (6.6%) | 0.164 |
| Death from respiratory disease | 10 (9.8%) | 13 (2.6%) | 0.002 |
| Death from other than lung cancer and respiratory disease | 9 (8.8%) | 14 (2.8%) | 0.026 |

IQR, interquartile range; CCI, Charlson comorbidity index; CT, computed tomography; GS, Goddard score; IP, interstitial pneumonia; SUV, maximum standardized uptake value; VC, vital capacity; FEV1, forced expiratory volume in one second; LY, lymphatic invasion; V, vascular invasion; PL, pleural invasion; LCNEC, large cell neuroendocrine carcinoma.
