# Supplementary material for: Prognosis of resected non-small cell lung cancer with pleural plaques on intrathoracic findings
Source: BMC Cancer. 2022 Apr 28;22:469. doi: 10.1186/s12885-022-09600-6 (PMC9052480; doi:10.1186/s12885-022-09600-6)
Supplement: Supplementary file 7 — Additional file 7: Table S4. Characteristics of clinical stage I patients with emphysema with Goddard score ≥5 points or interstitial pneumonia. [file 12885_2022_9600_MOESM7_ESM.docx]

**Supplemental Table 4. Characteristics of clinical stage I patients with emphysema with Goddard score ≥5 points or interstitial pneumonia**

| Variables | Plaques (+)  IP or emphysema (+)  n = 61 (28.2%) | Plaques (-)  IP or emphysema (+)  n = 155 (71.8%) | *P* value |
| --- | --- | --- | --- |
| Age (median, IQR) | 75 (70–80) | 74 (69–79) | 0.222 |
| Sex, Male | 58 (95.1%) | 119 (76.8%) | < 0.001 |
| Smoking history | 59 (96.7%) | 135 (87.1%) | 0.021 |
| Preoperative CT findings |  |  |  |
| Radiological emphysematous changes (GS ≥5 points) | 46 (75.4%) | 106 (68.4%) | 0.303 |
| Radiological IP findings | 36 (59.0%) | 86 (55.5%) | 0.637 |
| Whole tumor size (mm) (median, IQR) | 19 (14–24) | 20 (15–29) | 0.117 |
| Solid tumor size (mm) (median, IQR) | 17 (13–22) | 18 (13–24) | 0.250 |
| SUVmax | 6.3 (2.5–11.3) | 3.3 (1.8–7.0) | 0.003 |
| Pulmonary function |  |  |  |
| %VC (%) (median, IQR) | 93.7 (85.9–106.6) | 100.9 (86.1–111.7) | 0.049 |
| FEV1/FVC (%) (median, IQR) | 75.2 (67.9–80.4) | 75.2 (66.7–80.1) | 0.854 |
| Clinical stage |  |  | 0.061 |
| IA1 | 10 (16.4%) | 24 (15.5%) |  |
| IA2 | 29 (47.5%) | 66 (42.6%) |  |
| IA3 | 17 (27.9%) | 30 (19.4%) |  |
| IB | 5 (8.2%) | 35 (22.6%) |  |
| Surgical procedure |  |  | 0.492 |
| Wedge resection | 27 (44.3%) | 79 (51.0%) |  |
| Segmentectomy | 11 (18.0%) | 19 (12.3%) |  |
| Lobectomy | 23 (37.7%) | 57 (36.8%) |  |
| Pneumonectomy | 0 (0%) | 0 (0%) |  |
| Invasive characteristics |  |  |  |
| LY | 10 (16.4%) | 21 (13.6%) | 0.595 |
| V | 6 (9.8%) | 17 (11.0%) | 0.807 |
| PL |  |  | 0.974 |
| PL1 | 6 (9.8%) | 14 (9.0%) |  |
| PL2 | 2 (3.3%) | 7 (4.5%) |  |
| PL3 | 1 (1.6%) | 3 (1.9%) |  |
| Histology |  |  | 0.019 |
| Adenocarcinoma | 27 (44.3%) | 102 (65.8%) |  |
| Squamous cell carcinoma | 29 (47.5%) | 40 (25.8%) |  |
| Adenosquamous carcinoma | 1 (1.6%) | 8 (5.2%) |  |
| Sarcomatoid carcinoma | 0 (0%) | 1 (0.7%) |  |
| LCNEC | 3 (4.9%) | 3 (1.9%) |  |
| Lymphoepithelioma-like carcinoma | 1 (1.6%) | 0 (0%) |  |
| Mucoepidermoid carcinoma | 0 (0%) | 1 (0.7%) |  |
| Pathological stage |  |  | 0.988 |
| 0 | 2 (3.3%) | 5 (3.2%) |  |
| IA1 | 13 (21.3%) | 33 (21.3%) |  |
| IA2 | 16 (26.2%) | 41 (26.5%) |  |
| IA3 | 11 (18.0%) | 33 (21.3%) |  |
| IB | 13 (21.3%) | 29 (18.7%) |  |
| IIA | 1 (1.6%) | 1 (0.7%) |  |
| IIB | 4 (6.6%) | 12 (7.7%) |  |
| IIIA | 1 (1.6%) | 1 (0.7%) |  |
| Recurrence | 13 (21.3%) | 33 (21.3%) | 0.997 |
| Death from any cause | 27 (44.3%) | 37 (23.9%) | 0.004 |
| Death from lung cancer | 10 (16.4%) | 20 (12.9%) | 0.510 |
| Death from respiratory disease | 8 (13.1%) | 9 (5.8%) | 0.086 |
| Death from other than lung cancer and respiratory disease | 9 (14.8%) | 8 (5.2%) | 0.025 |

IQR, interquartile range; CCI, Charlson comorbidity index; CT, computed tomography; IP, interstitial pneumonia; GS, Goddard score; SUV, maximum standardized uptake value; VC, vital capacity; FEV1, forced expiratory volume in one second; LY, lymphatic invasion; V, vascular invasion; PL, pleural invasion; LCNEC, large cell neuroendocrine carcinoma.)
